# Chitosan-vancomycin hydrogel incorporated bone repair scaffold based on staggered orthogonal structure: a viable dually controlled drug delivery system

**DOI:** 10.1039/d2ra07828g

**Published:** 2023-01-25

**Authors:** Xiaohan Gao, Zexian Xu, Shangbo Li, Lidi Cheng, Dian Xu, Li Li, Liqiang Chen, Yaoxiang Xu, Zijian Liu, Yanshan Liu, Jian Sun

**Affiliations:** a The Affiliated Hospital of Qingdao University Qingdao 266000 China surgeonshan@outlook.com; b School of Stomatology of Qingdao University Qingdao 266000 China sunjianqdfy@qdu.edu.cn; c Dental Digital Medicine & 3D Printing Engineering Laboratory of Qingdao Qingdao 266000 China; d Shandong Provincial Key Laboratory of Digital Medicine and Computer-Assisted Surgery Qingdao 266000 China

## Abstract

In clinical practice, challenges remain in the treatment of large infected bone defects. Bone tissue engineering scaffolds with good mechanical properties and antibiotic-controlled release are powerful strategies for infection treatment. In this study, we prepared polylactic acid (PLA)/nano-hydroxyapatite (nHA) scaffolds with vertical orthogonal and staggered orthogonal structures by applying 3D printing technology. In addition, vancomycin (Van)-based chitosan (CS) hydrogel (Gel@Van) was loaded on the scaffold (PLA/nHA/CS-Van) to form a local antibiotic release system. The microstructure of the composite scaffold had high porosity with interconnected three-dimensional networks. The mechanical properties of the PLA/nHA/CS-Van composite scaffold were enhanced by the addition of CS-Van. The results of the water contact angle analysis showed that the hydrophilicity of the drug-loaded scaffold improved. In addition, the composite scaffold could produce sustained release *in vitro* for more than 8 weeks without adverse effects on the proliferation and differentiation of mouse embryonic osteoblasts (MC3T3-E1), which confirmed its good biocompatibility. During the *in vitro* antimicrobial study, the composite scaffold effectively inhibited the growth of *Staphylococcus aureus* (*S. aureus*). Therefore, our results suggest that the PLA/nHA/CS-Van composite scaffold is a promising strategy for treating infected bone defects.

## Abstract 2

The schematic diagram of preparing the composite scaffolds.

## Graphical abstract

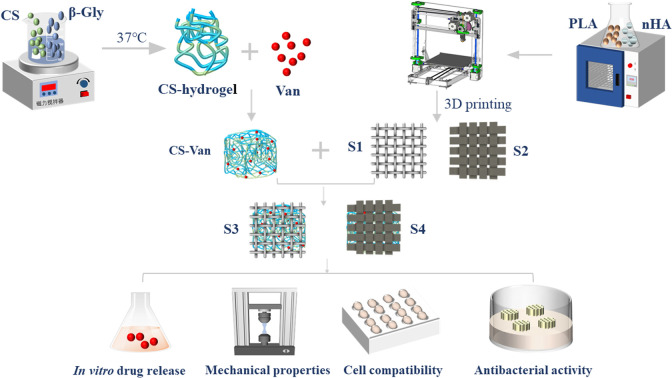

## 1. Introduction

Large oral and maxillary defects, which are mainly caused by severe trauma, tumor resection, and other factors, have been increasing in number.^1,2^ However, it is still difficult to reconstruct bone defects safely and effectively, especially in the presence of infection.^3^ The formation of bacterial biofilms reduces the activity of osteoblasts.^4,5^ The complexity of bone infections has led to changes in treatment strategies. Currently, infection control and local defect reconstruction are the two main principles for treating infected bone defects.^6,7^

Bones have inherent reconstructive potential.^8^ Moreover, autologous and allogeneic bone grafting, as well as guided osteogenesis, remains the choice in clinical practice, but these approaches have their limitations.^9,10^ Owing to their size and anatomical shape limitations, they have a low osteogenic potential and cannot be fully adapted to various clinical bone defects.^11,12^ Bone tissue engineering is dedicated to the application of bone regeneration, and scaffolds have attracted considerable attention as an important part of bone regeneration.^13,14^ Scaffolds for bone defects must have excellent performance, such as optimal mechanical properties, biocompatibility and controllable drug delivery capabilities.^15^ Polylactic acid (PLA) is the most commonly used synthetic polyester with biocompatibility and FDA approval.^16,17^ However, pure organic plastics used clinically are sub-optimal; thus, PLA is often modified with other materials to improve its performance.^18,19^ Hydroxyapatite (HA), a major component of bone minerals, is increasingly attractive for clinical and biological applications because of its high biocompatibility and low immunogenicity, but its utility is limited by its brittle mechanical properties, improving its properties with other materials is necessary. Studies have suggested that nHA combined with PLA form scaffolds with good mechanical properties and biocompatibility and are promising artificial bone graft materials.^20,21^ Controlled drug release can establish clinically relevant local drug concentrations, and many strategies have been developed in the construction of drug-loaded scaffolds for controlled release.^22–24^ Hydrogels are of particular interest in drug release owing to their ease of assembly from nature and biocompatibility.^25,26^ Chitosan (CS) is a naturally occurring polysaccharide, which is derived from chitin by deacetylation.^27^ Chitosan-hydrogels are three-dimensional networks that can absorb water or biological fluids in vast amounts, and drugs can be loaded into the hydrogels by physical absorption or encapsulation.^28^ Consequently, many studies have explored the application of CS-based biomaterials.^29,30^ Osteomyelitis is one of the most common maxillofacial-associated infections caused by *S. aureus*.^31,32^ Vancomycin (Van), a glycopeptide antibiotic, acts on Gram-positive bacteria mainly by blocking the synthesis of peptidoglycan and has less adverse effects on osteoblasts and bone regeneration.^33^ Therefore, we hypothesized that the combination of CS with Van would be a good controlled-release candidate for infection control and bone defect repair.^34,35^

The rapid development of digital medical technology has led to the widespread use of three-dimensional (3D) printing technology.^36^ The technology can produce scaffolds with reproducible tissue structure and mechanical properties that can precisely match complex bone defects in the maxillofacial region for custom filling.^37^

In this study, we used PLA and nHA as osteoconductive support materials to fabricate scaffolds with vertical orthogonal and staggered orthogonal structures. Moreover, CS-Van hydrogels were loaded into the scaffolds, forming a dually-controlled drug delivery system. The experimental results provide a scientific basis and technical support for individualized bone defect reconstruction.

## 2. Materials and methods

### 2.1. Materials

PLA was purchased from Sinopharm Chemical Reagent Co., Ltd. HA was purchased from Shanghai Aladdin Biochemical Technology Co., Ltd. CS was purchased from Shanghai Macklin Biochemical Technology Co., Ltd., and Van was purchased from Dalian Meilun Biotech Co., Ltd.

### 2.2. Preparation and characterization of CS-Van hydrogels

200 mg of CS powder was weighed and dissolved in 9 mL of glacial acetic acid solution (0.1% w/v). A mixed CS solution (2% w/v) was prepared; the resulting solution was stirred for 1.5 h until it completely became a yellow solution. Moreover, a β-glycerophosphate (56% w/v) solution was prepared and stored in a refrigerator at 4 °C for 15 min. 1 mL of β-glycerophosphate solution was added dropwise to the CS-solution at low speed, stirred for 15 min, and then the above-prepared solution was placed in a constant temperature water bath at 37 °C to form a hydrogel. Then, 5 mg of Van powder was dissolved in the CS hydrogel, producing a CS-Van hydrogel. The hydrogel samples were sprayed with gold particles after being dried, which was then observed for surface morphology using SEM (Hitachi JSM-7500F, Japan); FTIR spectra were collected using a Nicollet instrument (NEXUS-470, Thermo Nicolet, USA); XRD patterns were acquired to observe the crystal form by applying HR-XRD (D8 Advance, German) with a scanning angle ranging from 10° to 80°. Other elemental information, especially the corresponding valence states, was determined using XPS (Shimadzu Corporation, Japan).

### 2.3. Preparation and characterization of composite scaffolds

Representation of the scaffold virtual model was designed using Solidworks (Dassault Systemes, Waltham, MA, USA), indicating PLA/nHA vertical orthogonal scaffold (S1), PLA/nHA staggered orthogonal scaffold (S2), PLA/nHA/CS-Van vertical orthogonal scaffold (S3) and PLA/nHA/CS-Van staggered orthogonal scaffold (S4). After the design, scaffolds with various structures were fabricated using 3D printing. At a 4 : 1 (w:w) radio, 5 g of PLA and 1.25 g of HA powder were dispersed in 40 mL of 1,4-dioxane solution, frozen and prepared as a dry powder formulation, and printed using a 3D bionic printer cartridge. The scaffold size was 20 mm × 5 mm × 5 mm, with an in-plane aperture of 300 μm and a line width of 30 μm. The 3D printer was equipped with a nozzle of 250 μm diameter, a walking speed of 4 mm min^−1^, and a temperature controlled at 105 ± 5 °C. S1 and S2 were prepared by applying the method described above. S3 and S4 were prepared by creating a vacuum to force the CS-Van hydrogel to adsorb into S1 and S2. S1, S2, S3, and S4 were stored in a freezer at −80 °C, frozen overnight, and cut into approximately 0.5 × 0.5 cm slices. SEM (Hitachi JSM-7500F, Japan) was used to explore the internal structure of the observed scaffolds, and the compositional and elemental mapping images of the samples were analyzed using EDS (Oxford Instrument, Oxfordshire, UK).^38^

### 2.4. Mechanical properties

The mechanical properties were examined with a tensile-compression tester (Instron 5567, Instron, USA) at room temperature and a humidity of 30–70%. Then, compression loads were applied to the samples (cylindrical specimens, diameter: 5 mm and height: 1 mm) at a strain rate of 1 mm min^−1^ until each sample was compressed to 80% of its initial height.^39^

From these analyses, stress–strain curves were performed. Compressive strength was determined by the maximum point of the stress–strain curve, and the compressive modulus was obtained from the linear part of the curve. For each scaffold, Young's modulus was calculated from the slope of the stress–strain curve in the linear region of 5–10%.^40^ Experiments were performed to evaluate the changes after adding CS-Van to the scaffold. Three measurements were performed on each sample, and we used the average to calculate the mean and standard deviation.

### 2.5. Contact angle

The hydrophilicity of the scaffolds was evaluated by measuring the water contact angle using a water droplet shape analyzer (SSC, DC318P Color Camera, Japan). Three distilled water droplets were placed in each scaffold. When the liquid reached a resting state, images of the droplets were taken, and contact angles were measured using Image-J software.^41^ The mean and standard deviation values of the three replicate sample results were calculated.

### 2.6. *In vitro* release assay

10 mg of Van was accurately weighed and transferred to a volumetric flask containing PBS, and the total volume was adjusted to 100 mL using Van solutions at concentrations of 2, 4, 6, 8, 10 and 16 μg mL^−1^. The standard curve of Van was measured at (280 ± 2) nm using a UV-vis spectrometer (UV-vis, Shimadzu, Japan). A certain amount of CS-Van, S1 and S2 were loaded into dialysis bags and incubated in 20 mL of PBS (pH = 7.4) with continuous agitation. 2 mL of buffer was withdrawn at selected time intervals and replaced with an equal volume of fresh medium.^42^ Based on the calibration curve, the concentration of Van at each time was calculated, generating the Van cumulative release curve. The mean and standard deviation were calculated from three independent replicates.

### 2.7. Cell proliferation experiment

Scaffolds after sterilization and disinfection in each group were immersed in a 96-well plate, and 2 mL of cell culture medium (DMEM supplemented with 10% FBS + 1% penicillin/streptomycin) was added and then maintained in a humidified incubator (37 °C, 5% CO_2_). The extracts were obtained by incubation for about 24 h. MC3T3-E1 (ATCC, CRL-2594) cells harvested at the logarithmic growth phase were adjusted to a cell density of 5 × 10^4^ mL^−1^. The experiment was divided into 5 groups (control (simple medium), S1, S2, S3, and S4) with each set up in three replicate wells in each experiment. In 1 to 5 days of culture, a CCK-8 (10 μL) solution was added to the cell culture medium (100 μL) and incubated for 2 h. The optical density (OD) was then measured at 450 nm using an auto-microplate reader (BioTek, VT, USA). DAPI (Sigma-Aldrich) staining was performed to assess cell proliferation.

### 2.8. *In vitro* antibacterial activity assay

We employed the *S. aureus* strain derived from strain ATCC6538 as the primary strain for experiments and measured the radius of the inhibition zone to explore the antibacterial activity. *S. aureus* was grown in TSB medium (100 μL) and incubated at 37 °C for 24 hours. A single colony was inoculated into 5 mL of TSB. Then, the bacterial suspension was completed while adjusting the concentration to the 0.5 McFarland standard. A total of 100 μL of bacterial solution was spread onto each plate, and four groups (S1, S2, S3 and S4) of scaffolds (diameter: 10 mm, thickness: 3 mm) were removed from each plate and incubated at 37 °C for 24 h. Three parallel groups were set up in each group to measure the diameter of the antibacterial ring.

### 2.9. Statistical analysis

Data were processed by SPSS 26.0 (IBM, Japan), and *P* < 0.05 was considered a significant difference.

## 3. Results and discussion

### 3.1. Characterization and analysis of CS-Van hydrogels

**Fig. 1:**
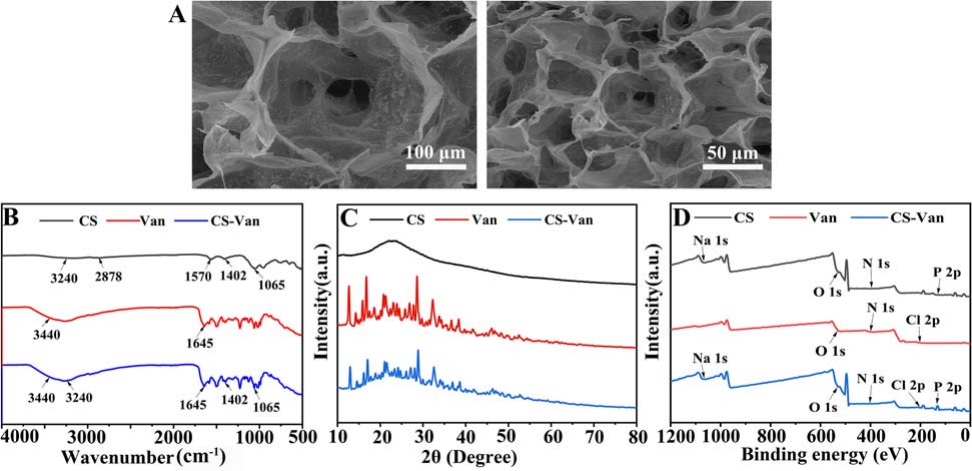
SEM images of CS-Van (A), FTIR spectra (B), XRD patterns (C) and XPS spectra (D) of CS, Van and CS-Van.

Fig. 1A shows a representative SEM image of the CS-Van hydrogel. A permeable porous structure was formed, facilitating the transport of water and macromolecules for cell growth.

As observed in the FTIR spectra (Fig. 1B), CS-Van hydrogels show characteristic absorption peaks of Van: 3440 cm^−1^ from O–H stretching vibrations and 1645 cm^−1^ from C=O. Although CS exhibits broad peaks at 3240 cm^−1^ and 2878 cm^−1^ corresponding to O–H and –NH_2_ stretching vibrations, no significant structural changes were found after the addition of Van. The FTIR results confirm the Van loading in CS-hydrogel. However, the limitation of FTIR is that it simply shows the physical co-mingling of Van and CS hydrogels and is unavailable for further accurate quantitative analysis.

The crystal structure was further characterized using XRD (Fig. 1C). The spectrum of the CS-Van hydrogel is equivalent to the sum of the CS broad reflection band and the sharp peaks of crystalline Van. The strong similarity between Van and CS-Van peaks suggests the presence of pure, solid and crystalline Van domains, reflecting the loading of Van into CS-hydrogel.

The XPS investigation (Fig. 1D) shows the main constituent elements of the CS-Van samples, C, N, O, Cl, and P, containing the total elemental composition of CS and Van, where a characteristic Cl 2p peak appears at 201.6 eV. Despite the presence of Van, there was no change in the structural changes in the CS. XPS results also demonstrate that CS and Van were successfully loaded.

### 3.2. Preparation and characterization of PLA/nHA/CS-Van

**Fig. 2:**
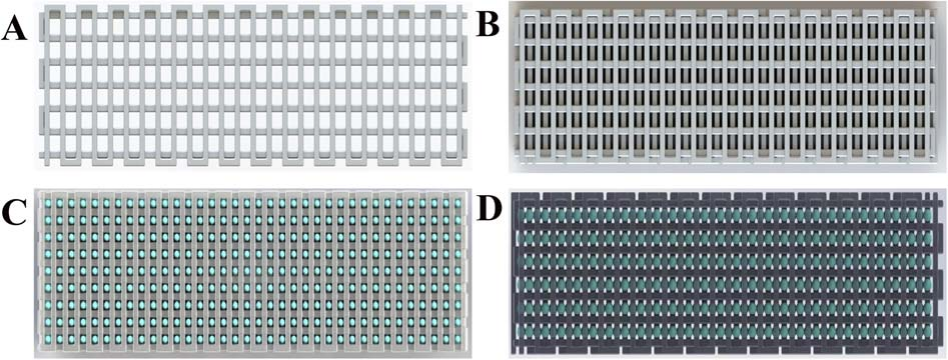
3D schematic diagram of S1 (A), S2 (B), S3 (C) and S4 (D).

The schematic diagram describing the scaffold (Fig. 2) was designed using computer-aided design modeling software (SolidWorks) and then manufactured by 3D printing, which helped advance the macro-structural assessment of the scaffold. As shown in the figure, two scaffolds with different structures were designed, S1 (Fig. 2A) and S2 (Fig. 2B); both have uniform pore sizes and high porosity that facilitate hydrogel loading. S3 (Fig. 2C) and S4 (Fig. 2D) reflect the binding state of the CS-Van hydrogel and scaffold with uniform distribution.

**Fig. 3:**
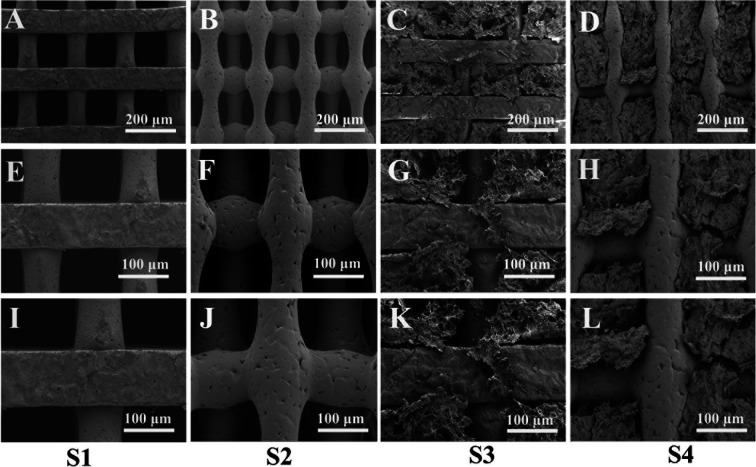
SEM imagine of S1, S2, S3, and S4 (A–D: 100×, E–H: 200×, I–L: 250×).

SEM was used to characterize the pore morphology and microstructure of the scaffolds (Fig. 3). The images (Fig. 3A and B) depict scaffolds with uniformly distributed pore structures and fairly smooth surfaces of S1 and S2 (Fig. 3E, F, I and J). Fig. 3C and D demonstrate that the CS-Van hydrogels are completely filled in the interconnected voids. The addition of hydrogels (Fig. 3G, H, K and L) appends the surface roughness of the scaffold and increases the formation of hydrophobicity, providing material for cell adhesion and survival. The results indicate that the composite scaffold can provide basic support for bone defects.

**Fig. 4:**
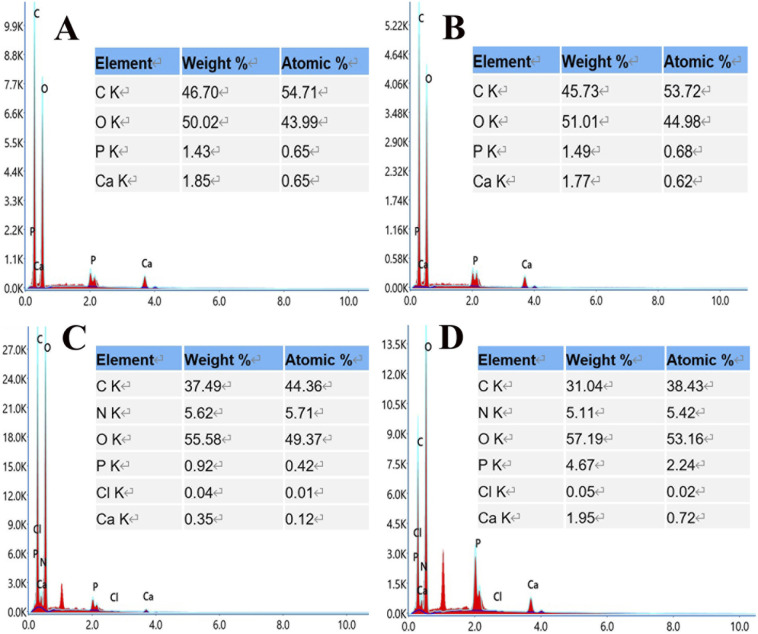
Element composition images of S1 (A), S2 (B), S3 (C), and S4 (D).

EDS analysis (Fig. 4) showed that S3 and S4 contain elements of C, O, N, Cl, P, and Ca supplied by PLA, nHA, CS, and Van, confirming the successful printing of the above substances at the molecular level.

**Fig. 5:**
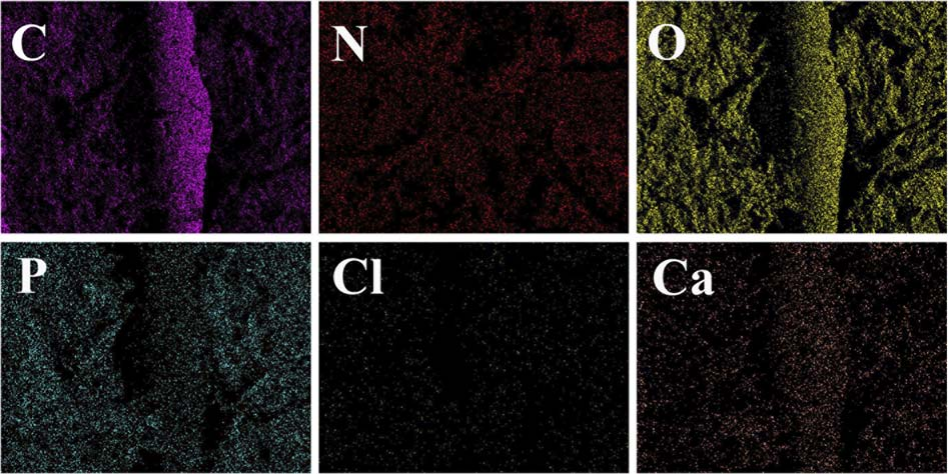
Elemental mapping images of C, O, N, Cl, P, and Ca in the S4 scaffolds.

Furthermore, elemental mapping (Fig. 5) shows a uniform distribution of elements on the surface of the composite scaffold without the admixture of other components. A control-released carrier was prepared based on the successful integration of CS-Van with the scaffold, providing infected bone defect further support.

### 3.3. Mechanical properties of the scaffold and contact angle analysis

**Fig. 6:**
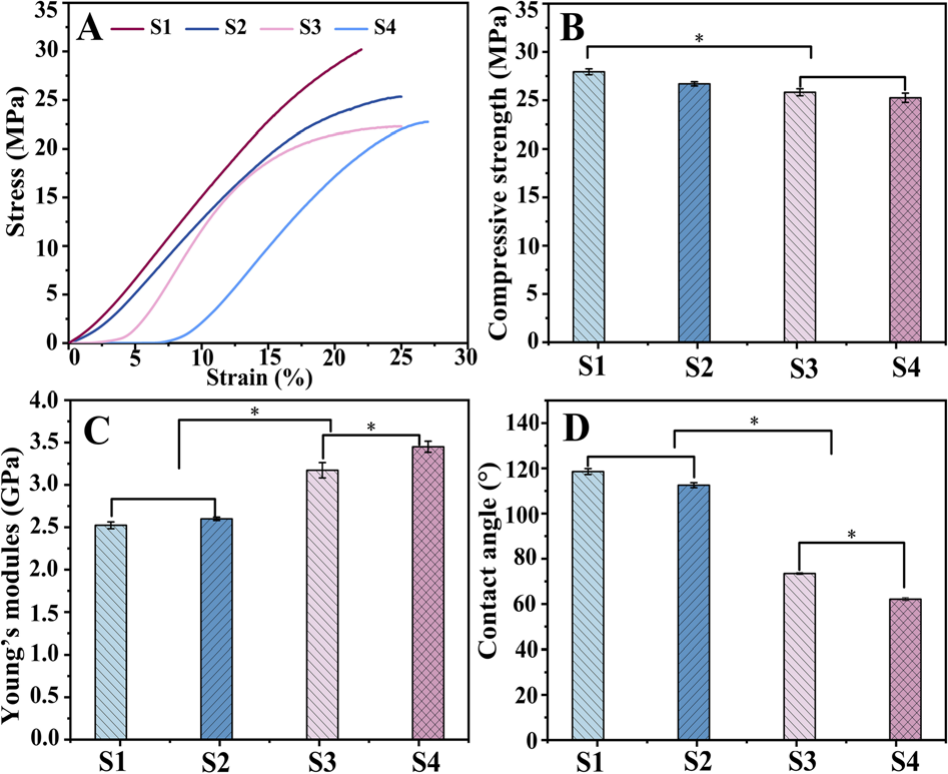
Compressive mechanical properties of scaffolds: stress–strain curve (A), compressive strength (B), Young's modulus (C), and contact angles (D) of scaffold.

Scaffolds for biomedical implants should withstand high contact loads, and Fig. 6 demonstrates the mechanical behavior of the scaffolds subjected to compression tests. Fig. 6A depicts the stress–strain curves of the S1, S2, S3 and S4 scaffolds. The mechanical testing dates were obtained to draw the following diagrams of compressive strength (Fig. 6B) and elasticity modulus (Fig. 6C).

The mean compressive strengths of S1 and S2 were (27.94 ± 0.31) MPa and (26.69 ± 0.21) MPa, respectively, which are slightly higher than those of S3 (25.82 ± 0.36) MPa and S4 (25.25 ± 0.48) MPa. This might be because during the bonding process of hydrogel with scaffold, water molecules can form hydrogen bonds to insert into the polymer chains and then weaken the molecular interactions, resulting in a decrease in scaffold strength.^43^ However, this difference is not clinically significant, and the composite scaffold can fully satisfy the mechanical properties of human bone.

The corresponding Young's modulus values were (2.52 ± 0.04) GPa (S1) and (2.6 ± 0.02) GPa (S2), while a significant increase in elasticity could be observed for S3 and S4 with (3.17 ± 0.09) GPa and (3.45 ± 0.06) GPa, respectively (*P* < 0. 05). This is because the hydrogels filled into the pores in the swollen state enhanced the elasticity of the material.

The analysis of the compression experiment showed better performance of drug-loaded scaffolds; simultaneously, S4 was superior to S3.

This study investigated only the effect of adding CS-Van to the scaffold on mechanical properties, and the influence of other factors is not considered. Most importantly, the composite scaffold provided fundamental mechanical support for bone defects.

In bone tissue engineering, the surface properties of materials are also important for biomedical applications. Wettability was assessed by the contact angle (Fig. 6D). The contact angles were significantly reduced (*P* < 0. 05) for S3 (73.45°) and S4 (62.11°) compared to S1 (118.55°) and S2 (112.53°), as the surface-exposed hydrogel could interact directly with the liquid. The high hydrophilicity of S4 is more conducive to cell adhesion and proliferation, exhibiting potential for the application of bone tissue engineering.

### 3.4. *In vitro* release assay

**Fig. 7:**
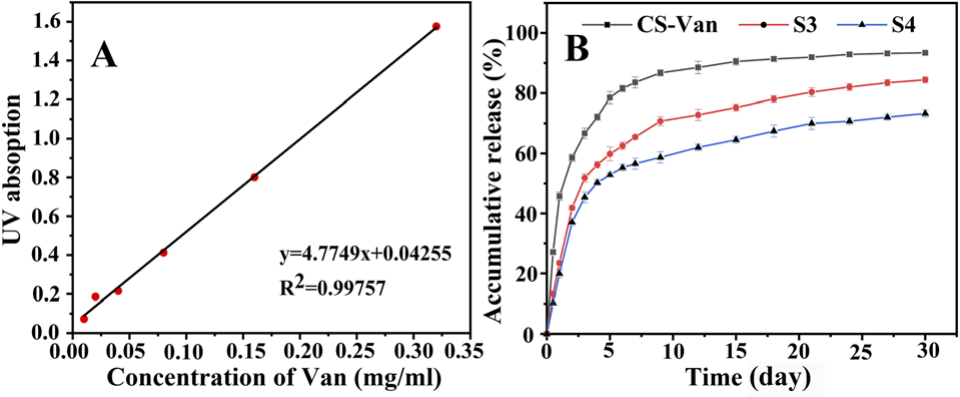
The standard curve of Van (A), *in vitro* release profiles (B) of CS-Van, S3 and S4.

As shown in Fig. 7B, the release of Van can occur for more than 8 weeks. After an initial burst release within the first 3 days, a more constant release rate followed. The slower release of S3 and S4 compared to CS-Van may be associated with effective drug loading and a favorable sustained-release system. By day 15, the cumulative release amount of CS-Van, S3 and S4 were (90 ± 3.3) %, (75 ± 4.1) % and (64.7 ± 3.6) %, respectively. On day 30, the amounts were (93.5 ± 3.6) %, (84.3 ± 4.3) %, and (73.8 ± 3.8) %, respectively. The results showed that the composite scaffolds had more excellent sustained-release ability than the CS-Van hydrogels. Moreover, the performance of S4 was better than S3, meaning that the dislocation structure feature is more favorable to the sustained release of drugs. This is essential for the S4 to exert continuous anti-infective ability in the infectious defect.

### 3.5. *In vitro* cell proliferation assay

**Fig. 8:**
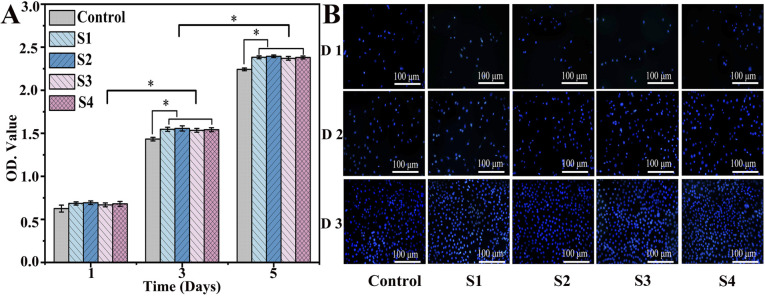
DAPI-stained nuclei images (A) and cell proliferation evaluation (B) of control, S1, S2, S3, and S4 groups for 1, 3, and 5 days.

The cytocompatibility of the composite scaffold is an important factor for further biomedical applications. On days 1, 3 and 5, cells were fixed, and the proliferation was assessed by DAPI staining (Fig. 8A). The cell density continued to increase for each group; cell counts in the S1, S2, S3, and S4 groups were significantly higher than those in the control groups after culturing (*P* < 0.05). As illustrated in Fig. 8B, the same result was also confirmed by CCK-8, which indicates that S3 and S4 have excellent biocompatible characteristics, supporting cell (MC3T3-E1) adhesion and proliferation. Moreover, cell viability in the S3 and S4 scaffolds was slightly higher compared to those in S1 and S2, demonstrating that the addition of CS-Van has no adverse effects on cell activity. However, the scaffolds provide a rougher surface caused by hydrogels, thus allowing for cell attachment and growth. These results highlight the potential value of S4 scaffolds as cell friendly materials for bone repair.

### 3.6. *In vitro* antimicrobial assay

**Fig. 9:**
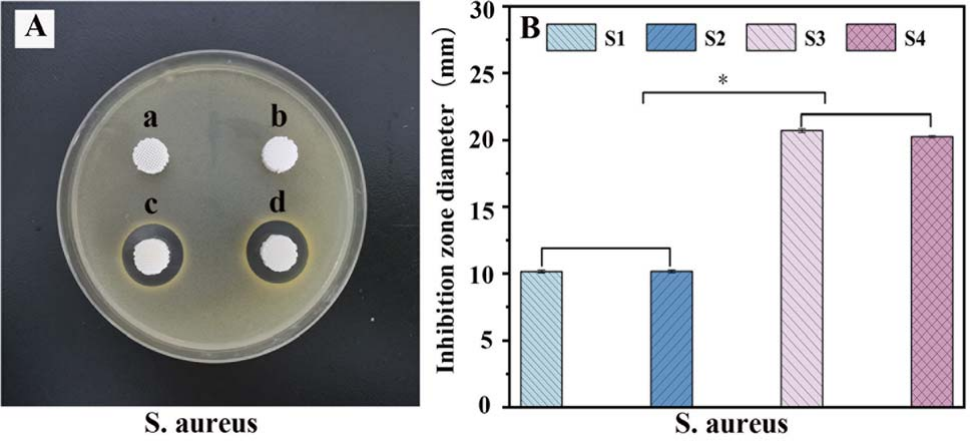
Antibacterial test (A) and the diameter of inhibition zone (B) for *S. aureus* with S1 (a), S2 (b), S3 (c) and S4 (d).

PLA/nHA/CS-Van scaffolds are expected to exhibit good antibacterial activity. To evaluate the antibacterial behaviors of the scaffold, a zone of inhibition (ZOI) test was carried out, with the diameters representing the antibacterial ability. Owing to the lack of effective target drugs, ZOI around the S1 and S2 scaffolds is weak or almost invisible. By contrast, the S3 and S4 scaffolds exhibit a pronounced inhibition area (Fig. 9A), indicating the antibacterial ability of Van released from the scaffold.

These results were further confirmed by histogram analysis (Fig. 9B) after scaffolds were co-cultured with bacteria for 24 h, which showed that the diameter of the S3 and S4 rings is larger than those of S1 and S2 (*P* < 0.05), implying better antibacterial properties. Moreover, S3 had a slightly larger diameter than S4, probably because S4 released fewer drugs than S3 over the same time frame. This proves that the S4 scaffold has a better sustained-release capacity on the other side, which is important for preventing infectious defects. The results suggest that S4 is a good candidate for bone tissue engineering.

## 4. Conclusions

In this study, we successfully synthesized a dually-controlled drug delivery system. The SEM shows that the scaffolds possess a highly porous and interconnected structure and that a rough surface is more conducive to the attachment of cells. The EDS results indicate that incorporating CS-Van into PLA/nHA scaffold and elements were uniformly dispersed. Compression experiments show that the compressive strength and Young's modulus of PLA/nHA/CS-Van staggered orthogonal scaffolds are compatible with human cancellous bone and provide a basis for cell attachment and growth of hydrophilicity. *In vitro*, drug-release experiments indicate that the Van in the staggered orthogonal scaffolds can be continuously released for more than 1 month, creating a local infection-controlled environment. In addition, the results of cell proliferation demonstrate that the PLA/nHA/CS-Van staggered orthogonal scaffolds have better biocompatibility and are promising for future clinical applications. Owing to the sustained release of the loaded antibiotics, PLA/nHA/CS-Van staggered orthogonal scaffolds exhibit remarkable antibacterial activity. The above results show that the 3D printed PLA/nHA/CS-Van staggered orthogonal scaffolds have great application potential in infection prevention and large segmental defect reparation.

## Author contributions

Conceptualization, S. L. and D. X.; writing – original draft preparation, Z. X.; writing – review and editing, L. L. and Y. X.; suggestions on revision, L. C. and Y. L.; funding acquisition, J. S. All authors have read and agreed to the published version of the manuscript. All authors have contributed substantially to the work reported.

## Conflicts of interest

There are no conflicts to declare.

## Supplementary Material
